# Der Mehrwert selbsthilfeorientierter Forschung in der Onkologie für Stakeholder in Deutschland

**DOI:** 10.1007/s00103-026-04185-y

**Published:** 2026-01-14

**Authors:** Christopher Kofahl, Stefanie Houwaart, Otmar Kodalle, Joachim Weis

**Affiliations:** 1 Institut für Medizinische Soziologie, Universitätsklinikum Hamburg-Eppendorf, Martinistr. 52, 20246 Hamburg, Deutschland; 2 BRCA-Netzwerk e. V. – Hilfe bei familiären Krebserkrankungen, Bonn, Deutschland; 3 partieval – Vermittlung partizipativer Kompetenzen, Prozessbegleitung und Evaluation im Bereich Gesundheit GmbH, Aachen, Deutschland; 4 Patientenbotschafter und -beirat, Universitätsklinikum Hamburg-Eppendorf, UCC Hamburg, Hamburg, Deutschland; 5 Universitätsklinikum Freiburg, Medizinische Fakultät, Tumorzentrum/CCC Freiburg, Professur für Selbsthilfeforschung, Universität Freiburg, Freiburg, Deutschland

**Keywords:** Selbsthilfe, Partizipation, Patientenbeteiligung, Patientenvertretung, Onkologische Forschung, Self-help, Patient organisation, Participation, Patient involvement, Oncological research

## Abstract

In der onkologischen Forschung wurden bedeutende Fortschritte erzielt, die die Behandlungsergebnisse verbessern. Dennoch zeigt sich zunehmend, dass die Perspektive der Patient:innen, insbesondere hinsichtlich Lebensqualität und psychischer Krankheitsbewältigung, stärker in den Fokus rücken muss. Der Artikel beleuchtet aus einer Multistakeholderperspektive die Integration von Patientenerfahrung und -wissen in die Krebsforschung mit dem Fokus auf selbsthilfeorientierte Forschung.

Im Rahmen von Patientenbeteiligung und -vertretung nimmt die Krebs-Selbsthilfe eine herausragende Stellung ein. Die Einbindung von Selbsthilfegruppen (SHG) und Selbsthilfeorganisationen (SHO) kann die Durchführbarkeit, Qualität und insbesondere die patient:innenseitige Relevanz onkologischer Studien erheblich verbessern. Der Grad der Partizipation von Mitgliedern aus SHG und SHO in Forschungsprojekten variiert stark – es lässt sich unterscheiden zwischen Forschung *ohne* Beteiligung der Selbsthilfe, Forschung *über, mit, durch* und *von* Selbsthilfe. Der partizipative und somit kooperative, gemeinsame und gleichberechtigte Ansatz gilt als der demokratischste und wird als optimal bewertet wird. Daraus ergeben sich Vorteile sowohl für direkt als auch indirekt beteiligte Interessengruppen wie Forschende, Patient:innen und Angehörige, Behandelnde und Versorgungseinrichtungen, Kranken- und Rentenversicherungen sowie die Gesellschaft. Selbsthilfeorientierte Forschung erfordert jedoch bestimmte Voraussetzungen. Im letzten Teil des Beitrags wird auf erfolgsfördernde Rahmenbedingungen sowie bestehende Barrieren und deren Überwindungsmöglichkeiten für eine gelingende Kooperation zwischen Wissenschaft und Selbsthilfe eingegangen.

## Abstract (en)

In recent years, oncological research has made significant progress improving treatment outcomes. Nevertheless, there is a growing recognition that greater focus must be placed on the patient’s perspective, particularly with regard to quality of life and psychological coping. This article examines the integration of patient experience and knowledge into cancer research from a multi-stakeholder perspective, with a focus on self-help-oriented research.

The article examines the integration of patient experience and knowledge into cancer research from a multi-stakeholder perspective, with a focus on self-help-oriented research. Cancer self-help plays a prominent role in patient participation and representation. The involvement of self-help groups (SHGs) and self-help organisations (SHOs) can significantly improve the feasibility, quality and, in particular, patient relevance of oncological studies. However, the degree of participation of members from SHGs and SHOs in research projects can vary greatly, from research without, to research with, or even research by self-help. The participatory and thus cooperative, joint and equal research represents the most democratic approach and is considered optimal. This results in various advantages for the directly and indirectly involved interest groups, such as researchers, patients and their relatives, healthcare professionals and care facilities, health and pension insurance funds, and society. However, self-help-oriented research is not without its prerequisites. The last part of the article discusses the conditions that promote success and the existing barriers to successful cooperation between research and self-help.

## Hintergrund

In den letzten Jahren wurden in der onkologischen Forschung bahnbrechende Fortschritte erzielt, die die Behandlungsergebnisse für Millionen von Menschen verbessern und die Sterblichkeit deutlich reduzieren, darunter innovative Ansätze wie CAR-T-Zelltherapien^1^, hochwirksame Rezeptorantagonisten, die Identifizierung prädiktiver Biomarker, die Entwicklung neuer Targets wie CALR-Mutationen^2^ oder neuer Wirkstoffkombinationen, insbesondere in der Immunonkologie [1]. Trotz oder gerade wegen dieser beeindruckenden Erfolge wächst die Erkenntnis, dass medizinisch-technischer Fortschritt allein den komplexen Herausforderungen einer Krebserkrankung nicht gerecht werden kann. Patient:innen fühlen sich häufig unzureichend informiert und an den sie betreffenden medizinischen Entscheidungen nicht ausreichend beteiligt [2]. Aspekte, die für die Betroffenen im Alltag von großer Bedeutung sind – wie Lebensqualität, krankheits- und therapiebedingte Einschränkungen oder die psychische Krankheitsbewältigung – rücken erst allmählich in den Fokus [3].

Dadurch erweitern sich die Fragestellungen in der Krebsforschung von dem, was medizinisch erstrebenswert ist, zu dem, was für ein gelingendes Leben mit und nach Krebs patient:innenseitig gewünscht und notwendig ist. Dieser Umstand begründet eine Forschung, in der die Perspektive der Betroffenen als integraler Bestandteil der Krebsforschung verstanden wird. In diesem Kontext kommt der organisierten Patient:innenschaft in ihren unterschiedlichen Formen – von regionalen Krebs-Selbsthilfegruppen (SHG) bis zu bundesweiten und internationalen Selbsthilfeorganisationen (SHO) – eine entscheidende Bedeutung zu.

Krebs-SHG und -SHO sind seit Jahrzehnten eine etablierte und unverzichtbare Säule der psychosozialen Versorgung von Krebspatient:innen und deren Angehörigen [4, 5]. Ihre Arbeit basiert auf dem Prinzip der gegenseitigen Hilfe auf Grundlage gemeinsamer Erfahrungen [6]. Die Aufgaben der organisierten Krebs-Selbsthilfe sind vielfältig und gehen weit über den reinen Erfahrungsaustausch hinaus. Sie umfassen im Wesentlichen 3 Kernbereiche:

1. *Psychosoziale Unterstützung:* SHG bieten emotionalen Beistand, helfen bei der Überwindung von Ängsten und geben Betroffenen und Angehörigen Mut und Verständnis [7].
2. *Informationsvermittlung:* Krebs-Selbsthilfe vermittelt Wissen über die Erkrankung, Behandlungsoptionen und den Umgang mit Nebenwirkungen, das u. a. über Broschüren, Websites, Podcasts oder durch geschulte Gruppenleiter:innen und Expert:innen verbreitet wird [8], (was jedoch keine ärztliche Konsultation ersetzen kann und soll).
3. *Interessenvertretung:* Krebs-SHO vertreten die Interessen von Krebserkrankten in sozial- und gesundheitspolitischen Gremien sowie in wissenschaftlichen Projekten, indem sie die Betroffenenperspektive einbringen [9].

Vor diesem Hintergrund unterstützt die Stiftung Deutsche Krebshilfe (DKH) Bundesverbände der Krebs-Selbsthilfe mit ca. 4,4 Mio. € pro Jahr (Stand 2024), womit diese u. a. personelle/ehrenamtliche Ressourcen und Strukturen bereitstellen. Strukturell fördert die DKH die Krebs-Selbsthilfe durch die Einrichtung des „Hauses der Krebs-Selbsthilfe – Bundesverband e. V.“ (HKSH-BV) als Dachorganisation von 12 Krebs-SHO sowie auch inhaltlich durch den Fachausschuss „Krebs-Selbsthilfe/Patientenbeirat“, der im Jahr 2004 etabliert wurde. Auch das Deutsche Krebsforschungszentrum (DKFZ) hat 2018 einen „Patientenbeirat Krebsforschung“ gegründet und realisiert seitdem Patientenbeteiligung^3^ in der Grundlagenforschung.

### Konzeptionelle Einordnung.

Selbsthilfeorientierte Forschung ist partizipative Forschung. Um ihren Mehrwert bzw. ihr Mehrwertpotenzial besser zu verstehen, erscheint eine konzeptionelle Abgrenzung verschiedener Partizipationsgrade sinnvoll. Die Unterscheidung zwischen Forschung *über, mit und durch* sowie *von* Selbsthilfe beschreibt eine evolutionäre Entwicklung der Selbsthilfebewegung – vom passiven Beobachtungsobjekt hin zu einem ermächtigten, aktiven Mitgestalter in der Forschungslandschaft ([10]; Tab. 1).

**Tab. 1:** Partizipationsgrade der Selbsthilfeforschung in der Onkologie

Stufe	Definition	Rolle der Selbsthilfe	Grad der Partizipation	Beispiele
Forschung *über* Selbsthilfe	Wissenschaftliche Untersuchung von SHG/SHO als Forschungsgegenstand	Passiver Forschungsgegenstand	Keiner bis gering	Untersuchung der Wirkungen und Wirksamkeit von SHG und SHO [7]
Forschung *mit und durch* Selbsthilfe	Kooperative Forschung, bei der Forschende und Selbsthilfe-Akteure partnerschaftlich zusammenarbeiten	Aktiver Kooperationspartner, Berater, Mitgestalter	Breites Spektrum von gering über mittel bis hoch	*ANKER-Projekt:* SH-Verbände gestalten Bedarfsanalyse für Angehörige mit [11, 12]
				*isPO-Projekt:* HKSH-BV ist Mitantragsteller und entwickelt Onkolotsen-Konzept mit [13, 14]
				*genomDE:* HKSH-BV und BRCA-Netzwerk gestalten die Vorbereitung des Modellvorhabens Genomsequenzierung nach § 64e SGB V mit
Forschung *von* Selbsthilfe	Von der Selbsthilfe initiierte, gesteuerte und/oder finanzierte Forschung	Initiator, Auftraggeber, Forschungstreibender	Geht über Partizipation hinaus bis „nicht mehr“ Partizipation	Promotionsstipendium der Deutschen Stiftung für junge Erwachsene mit Krebs [15]

*BRCA* Breast Cancer, hier genetisch bedingter Brustkrebs, *HKSH-BV* Haus der Krebs-Selbsthilfe – Bundesverband e. V., *SGB V* Sozialgesetzbuch V, *SH* Selbsthilfe, *SHG* Selbsthilfegruppe, *SHO* Selbsthilfeorganisation, *isPO* integrierte, sektorenübergreifende Psychoonkologie

Forschung *über* Selbsthilfe richtet sich vor allem auf Strukturen, Prozesse und Wirkungen von SHG und SHO. Typische Forschungsfragen sind: Welche Effekte hat eine SHG-Teilnahme auf Krankheitsbewältigung und Lebensqualität von Krebspatient:innen [16, 17]? Welche spezifischen Selbsthilfepotenziale haben besondere Gruppen, wie beispielsweise junge Erwachsene mit Krebs [18]? Wie ist Selbsthilfe strukturiert und welche Bedarfe bestehen für ihre Weiterentwicklung [19]?

Bei der Forschung *mit und durch* Selbsthilfe agieren SHG und SHO als gleichberechtigte Kooperationspartner im Forschungsprozess – idealerweise von der Entwicklung der Forschungsfrage über die Gestaltung der Studie bis zur Interpretation und Verbreitung der Ergebnisse [20]. Beispiele sind Projekte wie „Mutig, bunt, aktiv leben mit Metastasen“ initiiert von der Frauenselbsthilfe Krebs [21], das Projekt „Gesundheitskompetenz, Selbsthilfeaktivitäten und Versorgungserfahrung von Menschen mit Krebs“ (gesa-K) in Zusammenarbeit mit dem HKSH-BV [22], das ANKER-Projekt zu psychosozialen Belastungen und Unterstützungsbedarf von Angehörigen krebskranker Menschen [11] oder das isPO-Projekt (integrierte, sektorenübergreifende Psychoonkologie). Das Konzept der „isPO-Onkolotsen“ wurde maßgeblich vom HKSH-BV mitentwickelt, um den realen Bedürfnissen der Patient:innen zu entsprechen [23]. Die Krebs-Selbsthilfe ist bei diesen Projekten inhaltlicher Impulsgeber und Praxispartner der Wissenschaftler:innen zur Ausgestaltung der Konzepte und Forschungsfragen sowie bei der Beurteilung und Diskussion der Forschungsergebnisse. Darüber hinaus fungiert Selbsthilfe auch als Multiplikatorin in der Gewinnung von Studienteilnehmenden sowie der Verbreitung und Verstetigung der Ergebnisse innerhalb ihrer eigenen Strukturen.

Auf der über Partizipation hinausgehenden Stufe, der Forschung *von* der Selbsthilfe, werden SHO selbst zu Initiatoren, Steuerungsinstanzen oder gar Förderern von Forschung. Hier definieren SHO eigenständig Forschungsagenden, die meist aus den Problemen und Fragestellungen ihrer Mitglieder abgeleitet sind, akquirieren Finanzmittel, beauftragen wissenschaftliche Einrichtungen mit der Durchführung oder führen Projekte in Eigenregie durch. Beispiele hierzu finden sich eher bei den großen, mit hauptamtlichen Strukturen ausgestatteten SHO wie der Deutschen Rheuma-Liga [24], der Deutschen Multiple Sklerose Gesellschaft [25] oder der Bundesarbeitsgemeinschaft Selbsthilfe von Menschen mit Behinderung, chronischer Erkrankung und ihren Angehörigen (BAG SELBSTHILFE), z. B. mit der Long-COVID-Plattform [26].

Selbsthilfeorientierte Forschung ist sowohl patient:innenorientiert als auch partizipativ. Letzteres meint die aktive und partnerschaftliche Einbeziehung relevanter Interessengruppen (Stakeholder). Dazu zählen – neben den Patient:innen und ihren Angehörigen – Mitglieder des Versorgungssystems, der Leistungs- und Entscheidungsträger sowie Forschungsförderer, idealerweise über den gesamten Forschungsprozess [27]. Partizipative Forschung wird als ein zentraler Aspekt der Patient:innenzentrierung und als ein Schlüsselelement für eine qualitativ hochwertige Gesundheitsversorgung angesehen [28], was durch hochrangige politische Initiativen unterstrichen wird. Die *Nationale Dekade gegen Krebs* hat die Stärkung der Patientenbeteiligung in der Krebsforschung zu einem ihrer wichtigsten Ziele erklärt [29]. Mit der „Allianz für Patientenbeteiligung in der Krebsforschung“ im Jahr 2022 wurde ein starkes Signal gesetzt, die Bedürfnisse der Betroffenen in den Mittelpunkt zu stellen und die Patient:innenpartizipation als neuen Standard in der deutschen Forschungslandschaft zu etablieren [30].

### Selbsthilfe-Akteure in der Patientenbeteiligung und -vertretung.

Selbsthilfeorientierte Forschung fokussiert auf *kollektive* Patientenbeteiligung. Diese findet in der Regel über organisierte und strukturierte Patienten*vertretungen* statt, also primär über Mitglieder von SHG und SHO als Sprecher:innen für ihre Gruppe oder Organisation [31]. Patientenvertretende sprechen nicht für sich selbst, sondern für ihr Kollektiv, d. h. ihre SHG, SHO oder weitere Gleichbetroffene. Die meisten Akteure, die patientenseitig in institutionalisierten Gremien wie dem Patientenbeirat des Deutschen Krebsforschungszentrums (DKFZ) oder dem Patientenforschungsrat des Nationalen Centrums für Tumorerkrankungen (NCT) Heidelberg mitwirken, sind überwiegend engagierte und vernetzte Vertreter:innen der organisierten Krebs-Selbsthilfe. Große onkologische Zentren wie die von der DKH geförderten Comprehensive Cancer Centers (CCC) haben inzwischen feste Kooperationsstrukturen mit Dutzenden von SHO und SHG und institutionalisieren den Austausch beispielsweise über runde Tische der Selbsthilfe [z. B. 32], Patientenbeiräte [z. B. 33] oder Patientenbotschafter:innen [z. B. 34].

**Abb. 1:**
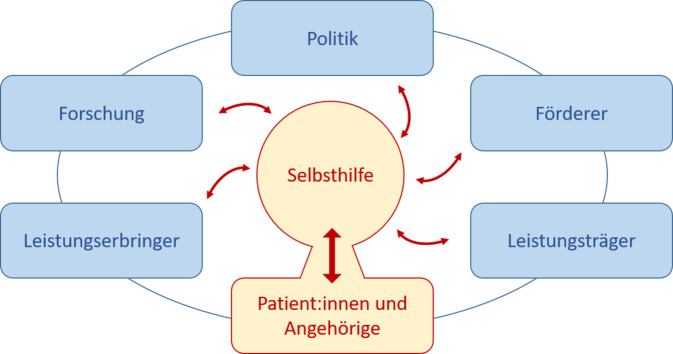
Krebs-Selbsthilfe als mediierende Instanz zwischen Patient:innen, ihren Angehörigen und Stakeholdern. Selbsthilfe = Selbsthilfegruppen, -organisationen, -initiativen oder -netzwerke

Selbsthilfeorientierte Forschung berücksichtigt und integriert die Potenziale der Krebs-Selbsthilfe, um eine breitere und größere Patient:innenorientierung zu bewirken. Die Selbsthilfe fungiert hier als eine mittelnde Instanz zwischen Betroffenen, Versorgenden, Leistungsträgern und Politik (Abb. 1). Forschungsergebnisse erreichen dadurch eine größere Durchdringung und Verbreitung in den jeweiligen Stakeholder-Gruppen.

## Fragestellung des Artikels und Vorgehensweise

Dieser Beitrag verfolgt das Ziel, den Mehrwert einer systematischen, selbsthilfeorientierten Forschung in der Onkologie zu beschreiben und zu analysieren. Anhand verschiedener Beispiele wird dargelegt, wie die Integration der Betroffenenexpertise durch die Krebs-Selbsthilfe die onkologische Forschung qualitativ verbessern, ihre Relevanz erhöhen und letztlich zu einer besseren Versorgung führen kann. Die Analyse erfolgt basierend auf der Expertise der Autor:innen^4^ und beleuchtet den Nutzen für verschiedene Stakeholder (Tab. 2).

**Tab. 2:** Mehrwert selbsthilfeorientierter Forschung für verschiedene Stakeholder in der Onkologie

Stakeholder	Art des Mehrwerts	Begründung/Mechanismus
Forschende	Qualität, Relevanz, Effektivität	Identifikation relevanter Forschungsfragen; patientenfreundlichere Studiendesigns; höhere patientenseitige Akzeptanz; höhere Mitwirkungsbereitschaft [35]
In Forschung einbezogene Patient:innen/Angehörige	Empowerment, passgenauere Versorgung	Forschung fokussiert auf patientenrelevante Endpunkte (z. B. Krankheitserleben, Lebensqualität); Einbringen des eigenen und kollektiven Erfahrungswissens, Wertschätzung der Erfahrungskompetenz, höhere Selbstwirksamkeit und Handlungsfähigkeit [36]
Behandelnde/Versorgungseinrichtungen	Praxisrelevanz, verbesserte Kommunikation und Prozesse, Ganzheitlichkeit	Ergebnisse sind direkt anwendbar; höhere Beratungskompetenz der Behandelnden (alltagsnah, patientenrelevant, systemisch); bedarfsgerechtere SDM-Tools; Clearing-Funktion der Selbsthilfe unterstützt und entlastet Personal [37, 38]
Kranken- und Rentenversicherung	Gesundheitsökonomie, Bedarfsgerechtigkeit, Steuerung	Potenzial zur Vermeidung von Über‑, Unter- und Fehlversorgung, inklusive der Folgekosten; Grundlage für innovative Versorgungsverträge (z. B. mit Onkolots:innen); Korrektur von Fehlsteuerungen (z. B. unnötige Frühberentung, inadäquate Hilfs- und Pflegemittel); Nachhaltigkeit von Behandlung und Rehabilitation [39]
Förderer	An der Zielgruppe orientierter Mitteleinsatz	Der Einsatz meist steuerfinanzierter oder gespendeter Forschungsgelder erfährt eine höhere demokratische Legitimation
Gesellschaft/Politik	Demokratisierung, Bürgerbeteiligung, Anerkennung verschiedener Wissensformen, Akzeptanz, Vertrauen	Forschung wird multiperspektivischer und besser übertragbar in die Lebensrealitäten; Stärkung des Vertrauens in das Gesundheitssystem durch wirkungsvolle Einbeziehung vielfältiger und vor allem marginalisierter Gruppen [40]

*SDM* Shared Decision Making = partizipative Entscheidungsfindung

Zur Eingrenzung der hohen Komplexität der Thematik, welche dem Gebiet des „Patient and Public Involvement“ (PPI; [41]) zuzuordnen ist, richtet sich der Fokus auf Deutschland. PPI ist in anderen Gesundheitssystemen bereits früher als in Deutschland zu einer drängenden Frage geworden, weshalb die Literatur hierzu inzwischen sehr umfangreich ist. Das Vereinigte Königreich nimmt hier mit seinem National Institute for Health and Care Research (NIHR) eine Vorreiterrolle ein [42], insbesondere durch dessen „UK Standards for Public Involvement“ [43]. Daneben existieren unzählige weitere Definitionen, Konzeptionen, Modelle, Regularien oder Verfahren. Der systematische Review von Greenhalgh et al. zeigt deutlich, dass es keine „One-size-fits-all-Lösung“ gibt (und diese auch nicht sinnvoll ist) und deshalb zu empfehlen sei, sich in Projekten und Studien mithilfe existierender Module sein jeweils eigenes Rahmenwerk zusammenzustellen [44].

Eine auf die Fragestellung und auf Deutschland eingegrenzte PubMed-Recherche^5^ ergab 68 Treffer. Hierunter befinden sich vereinzelt konzeptionelle Arbeiten, während die Mehrheit der Artikel Studien abbildet, in denen Patient:innen, Patient:innenvertretende oder -fürsprechende („advocates“) involviert waren. Hinsichtlich einer expliziten Einbindung von Selbsthilfe- bzw. Patientenorganisationen, teils auch in Co-Autorenschaften der Selbsthilfevertretenden, ließen sich letztlich nur 8 Artikel zuordnen [39, 45–51]. Selbsthilfeorientierung in der Forschung stellt einen besonderen, bislang jedoch wenig thematisierten PPI-Ansatz dar. Auf diesen wollen wir mit unserem Beitrag eingehen.

## Selbsthilfeorientierung als Mehrwert in der onkologischen Forschung

Der wesentliche Vorteil einer Integration der *Selbsthilfe* in die onkologische Forschung (in Abgrenzung zur *Patientenbeteiligung* in der Forschung) besteht zum einen darin, dass für Krebserkrankte relevante Themen, die nicht nur auf den individuellen Erfahrungen *einzelner* Patient:innen basieren, bereits „voraggregiert“ (z. B. durch Selbsthilfe-Tagungen, Workshops, Arbeitsgruppen der SHO, Mitgliederumfragen) in die Studienkonzeption einfließen können (Input). Zum anderen fließen die gewonnenen Ergebnisse über die Kanäle der Selbsthilfe zurück an die potenziell involvierten Stakeholder (Output), idealerweise in Form gemeinschaftlicher Publikationen, Handlungsempfehlungen, Patient:innen-Leitlinien etc. Selbsthilfeorientierte Forschung schafft so eine symbiotische Wertschöpfungskette, in der der Vorteil für einen Stakeholder auch einen Nutzen für einen anderen generiert.

Diese „Win-win-Situation“ führt zu einem positiven Kreislauf, der das Gesamtsystem der onkologischen Versorgung und Forschung verbessert. Identifiziert eine SHG oder SHO beispielsweise eine relevante Forschungsfrage zur Bewältigung von therapiebedingter Fatigue, entsteht zunächst ein Mehrwert für die Patient:innen, da ein für sie bedeutsames Problem adressiert wird. Forschende profitieren von einer relevanten Forschungsfrage und einer höheren Mitwirkungsbereitschaft für ihre Studie. Praxisnahe Ergebnisse, wie etwa ein evaluiertes Programm zum Fatigue-Management, bieten Behandelnden und Versorgungseinrichtungen anwendbare Lösungen zur Verbesserung der Versorgung. Dies kann zu nachhaltigeren Rehabilitationserfolgen und potenziell geringeren Folgekosten führen, was wiederum einen Mehrwert für die Krankenkassen und Rentenversicherungen darstellt. Die SHO, die den Prozess angestoßen und begleitet hat, kann die gemeinsam gewonnenen Ergebnisse als Forschungspartnerin innerhalb ihrer Community verbreiten, ihren Bekanntheitsgrad erhöhen und weitere Mitglieder gewinnen, die idealerweise von der Mitgliedschaft profitieren, wodurch der Nutzen für alle maximiert wird. Tab. 2 gibt eine Übersicht über die unterschiedlichen Formen des Mehrwerts.

Vor dem Hintergrund der hier teils theoretischen Darstellung ist kritisch anzumerken, dass es zu wenige Untersuchungen über die Beteiligung von SHG/SHO in der Forschung bzw. im Forschungsprozess gibt. Selbsthilfeorientierte Forschung kann im Wissenschaftssetting nur gelingen, wenn ausreichend gute Informationen darüber vorliegen, wie sie von allen Beteiligten am besten umgesetzt werden kann und welche Rahmenbedingungen dafür erforderlich sind.

### Mehrwert für Forschende und Wissenschaft.

Für die Wissenschaft selbst sind die Kooperation mit der Selbsthilfe und die Einbeziehung von Patientenvertreter:innen kein Akt der Philanthropie, sondern eine strategische Investition in die Qualität und Effizienz der eigenen Arbeit. Durch den direkten Einbezug von Betroffenen wird sichergestellt, dass die Forschung *bedarfs- und bedürfnisgerecht* ist. Krebs-Patient:innen und ihre Angehörigen sind die einzigen Menschen, die die gesamte onkologische Versorgung von Diagnosestellung über Behandlung und Reha bis zur Nachsorge und/oder ggf. palliativen Versorgung mit all ihren Schnittstellenproblemen, Versorgungssektoren, administrativen und rechtlichen Herausforderungen an Leib und Seele erfahren haben. Dadurch sind sie die maßgeblichen Akteure in der „letzten Meile“ der Versorgung [52]. Selbsthilfe greift dieses als kollektives Erfahrungswissen auf und gibt dies an Forschende weiter.

### Mehrwert für Selbsthilfegruppen und -organisationen.

Die aktive Mitwirkung in Forschungsprojekten stellt für die SHG und SHO eine Form der Interessenvertretung und damit eine Umsetzung ihrer Ziele dar. Sie ist zudem ein Motor für ihre Weiterentwicklung und ggf. Professionalisierung. Die Mitarbeit in Gremien, bei der Leitlinienentwicklung oder in Forschungsprojekten wird neben der Erfahrungsexpertise durch vielfältige Fertigkeiten und Kompetenzen erleichtert, die von wissenschaftlichem Grundverständnis bis hin zu analytischen und strategischen Fähigkeiten reichen. Dies treibt einen Befähigungs- und Professionalisierungsprozess innerhalb der Selbsthilfe an. Dazu wurden verschiedene Maßnahmen zur Fortbildung von Patientenvertretenden entwickelt wie das von der DKH geförderte *Zentrum für Kompetenzentwicklung in der Krebs-Selbsthilfe (ZfK KSH)* an der Universität Freiburg, die vom NCT entwickelte/etablierte *Patienten-Experten Akademie für Tumorerkrankungen (PEAK)* am DKFZ in Heidelberg oder das *Patientenkompetenzzentrum Nord* der Universitären Cancer Centers (UCCs) Hamburg und Schleswig-Holstein.

Die Anerkennung des kollektiven Erfahrungswissens als eine dem akademischen Fachwissen komplementäre Wissensform stärkt die SHG und SHO in ihrer Rolle als Forschungspartner und politischer Akteur. Mit ihrer Expertise bringen sich Selbsthilfemitglieder stellvertretend für die Patient:innenschaft, innerhalb derer sie vernetzt sind, selbstwirksam in Forschungsprojekte und den politischen Diskurs ein. Sie gestalten über die Forschung eine passgenauere Versorgung und erzielen mit ihrem politischen Engagement eine Verbesserung der rechtlichen und gesellschaftlichen Rahmenbedingungen für Betroffene und ihre Angehörigen.

### Mehrwert für Patient:innen und Angehörige.

Der unmittelbarste und wichtigste Mehrwert entsteht für die Betroffenen selbst. Selbsthilfeorientierte Forschung legt den Fokus auf relevante und besondere Lebensthemen, welche direkt aus der Lebenswirklichkeit der Betroffenen stammen. Beispiele sind die psychosoziale Belastung von Angehörigen, der informierte Umgang mit Komplementärmedizin oder die spezifischen Herausforderungen junger Erwachsener mit Krebs. Die Beteiligung am Forschungsprozess und die Nutzung der daraus entstehenden Produkte (z. B. verständliche Informationsmaterialien, Entscheidungshilfen) stärken das Empowerment und die Gesundheitskompetenz der Patient:innen [16].

### Mehrwert für Behandelnde und Versorgungseinrichtungen.

Auch für Ärzt:innen, Pflegende und das Management von Krankenhäusern und Praxen bietet die selbsthilfeorientierte Forschung handfeste Vorteile. Mitglieder von SHG und SHO helfen, optimale Instrumente für Information, Aufklärung und Entscheidungsfindung zu entwickeln. Eine starke und gut informierte Selbsthilfe kann zudem eine wichtige „Clearing-Funktion“ für Versorgungseinrichtungen übernehmen. Indem SHG Peer-Support leisten und bei der Orientierung im komplexen Gesundheitssystem unterstützen, entlasten sie das klinische Personal. Um ein Beispiel zu geben: Das isPO-Projekt hat diese Funktion mit den Onkolots:innen institutionalisiert. Diese fortgebildeten, ehemals selbst betroffenen Ehrenamtlichen fungieren als eine Art „Tourist-Information im Neuland Krebs“ und weisen den Patient:innen den Weg zu qualitätsgesicherten Unterstützungsangeboten [53].

### Mehrwert für Krankenkassen und andere Kostenträger.

Für Krankenkassen und andere Kostenträger, wie die Rentenversicherung, ist selbsthilfeorientierte Forschung aus strategischer und gesundheitsökonomischer Sicht von großem Interesse, denn besser informierte Patient:innen tragen dazu bei, Über‑, Unter- und Fehlversorgung zu reduzieren. Die Vermeidung unnötiger oder unerwünschter Behandlungen ist ein explizites Ziel von Förderrichtlinien im Rahmen der Nationalen Dekade gegen Krebs [54]. Ein besseres Management von Nebenwirkungen kann zudem teure Krankenhausaufenthalte oder Notfallbehandlungen verhindern. Dies gibt den Krankenkassen eine evidenzbasierte Grundlage für innovative und bedarfsgerechte Versorgungsangebote. Obwohl harte ökonomische Daten rar sind, scheint das Potenzial für eine effizientere und gleichzeitig bedarfsgerechtere Ressourcenallokation evident.

### Gesellschaftlicher und politischer Mehrwert.

Die Beteiligung von an Krebs erkrankten Bürger:innen am Forschungsprozess macht diesen vielfältiger und demokratischer. Die „Schwarmintelligenz“ der Selbsthilfe-Community, die sich u. a. in Chat-Foren, Blogs und Social-Media-Gruppen äußert, stellt ein großes, oft ungenutztes Sammelsurium an Erfahrungen, Fragen und Meinungen dar, welches strukturiert aufbereitet und entsprechend an die relevanten Stakeholder kommuniziert werden kann. So hat beispielsweise einer der Autor:innen in seiner Rolle als Selbsthilfevertreter die von einer bundesweiten Facebook-Gruppe aufbereiteten persönlichen Erfahrungsberichte von Speiseröhrenkrebs-Betroffenen regional weiterverbreitet damit diese in die ärztliche Aufklärung und Beratung im Rahmen der onkologischen Nachsorge Eingang finden. In einem anderen Beispiel nutzten Forschende das Selbsthilfeforum der Frauenselbsthilfe Krebs, in dem sich Patientinnen online austauschen, um patientinnenseitige Einblicke in das Selbstmanagement von Nebenwirkungen zu erhalten, was zahlreiche neue oder bislang unterschätzte Aspekte zu Tage brachte [55].

Durch Einbeziehung verschiedener, vor allem auch marginalisierter Gruppen kann das nicht immer gegebene Vertrauen in die Wissenschaft erhöht werden. Dazu gehört allerdings eine adressat:innenorientierte Berichterstattung über die selbsthilfeorientierte Forschung, die neben den klassischen wissenschaftlichen Publikationen auch über sogenannte graue Literatur (adressat:innengerechter: „bunte“ Literatur) sowie die Kanäle der beteiligten SHG und SHO erfolgt.

## Rahmenbedingungen und Voraussetzungen für eine gelingende Kooperation

Damit selbsthilfeorientierte Forschung ihr volles Potenzial entfalten kann, müssen bestimmte Rahmenbedingungen erfüllt sein. Eine erfolgreiche Kooperation ist kein Selbstläufer, sondern erfordert eine wertschätzende Haltung, bewusste Gestaltung der Zusammenarbeit, unterstützende institutionelle Strukturen und einen klaren politischen Willen.

### Faktoren für gelingende Zusammenarbeit.

Besonders bedeutsam für eine partnerschaftliche Zusammenarbeit zwischen Forschung und Selbsthilfe ist eine *frühzeitige und kontinuierliche Einbindung*. Selbsthilfevertretende sollten nicht am Rande und nur aus Gründen des Abhakens von Förderkriterien in ein bereits fertiges Projektkonzept einbezogen werden („Pseudobeteiligung“). Ihre Beteiligung sollte bereits bei der Auswahl und Formulierung der Forschungsfrage beginnen und sich durch alle Phasen des Forschungsprozesses ziehen, von der Planung über die Durchführung bis hin zur Dissemination der Ergebnisse. Nur so kann ihre Expertise umfänglich wirksam werden.

Des Weiteren sollte die Kooperation von *gegenseitigem Respekt und der Anerkennung der jeweils anderen Expertise* geprägt sein. Forschende sollten die Betroffenenperspektive als gleichwertige, ihr eigenes Wissen ergänzende Wissensquelle anerkennen, während Selbsthilfevertreter:innen wissenschaftliche Methoden und Rahmenbedingungen und die damit verbundene Forschungslogik respektieren sollten.

Das Engagement in der Selbsthilfe in der Forschung sowie der Patientenvertretung ist eine anspruchsvolle Aufgabe, die Zeit, Energie und auch finanzielle Ressourcen erfordert. Eine *angemessene finanzielle Anerkennung* im Sinne von Übernahme der Reisekosten, von pauschalen Aufwandsentschädigungen und gegebenenfalls auch die Kompensation von Verdienstausfällen ist daher nicht nur eine Frage der Fairness, sondern oft eine notwendige Voraussetzung für eine nachhaltige und inklusive Beteiligung. Die SHO sind in Ergänzung zu Mitgliedsbeiträgen und Spenden auf finanzielle Unterstützung insbesondere durch die Selbsthilfeförderung nach SGB V § 20h angewiesen, haben in der Regel also keine eigenen Mittel, mit denen sie eine Forschungsbeteiligung bestreiten können. Die betreffenden Förderorganisationen sollten in Erwägung ziehen, finanzielle Ressourcen für Vertreter:innen aus den SHO und SHG für die Mitwirkung bei der Entwicklung von Projekten und deren Antragstellungen vorzusehen.

### Barrieren und Ansätze zur Überwindung.

Bei manchen Forschenden herrschen noch Skepsis und Unsicherheit oder gar Vorbehalte, inwieweit Betroffene ohne wissenschaftliche Ausbildung über genügend Fachwissen verfügen, um einen sinnvollen Beitrag, insbesondere in der methodisch komplexen Grundlagenforschung, leisten zu können [29]. Es besteht zudem Sorge, dass Prozesse verlangsamt oder verkompliziert werden, denn in der Tat ist die Einbindung von Patienten- und Selbsthilfevertretenden mit zusätzlichen Aufwendungen verbunden. Für die Forschenden existieren daher inzwischen verschiedene Hilfestellungen, um sie zur Kooperation mit Patienten- und Selbsthilfevertretenden zu befähigen [z. B. 3, 20, 56, 57]. Allein dies zeigt bereits, dass der in den meisten Förderauflagen mittlerweile übliche Zusatz „Patientinnen und Patienten sind zu beteiligen“ nicht hinreichend ist, um diese Forderung auch angemessen zu erfüllen.

Hürden existieren auch seitens der Selbsthilfe-Akteure. Die gesundheitlichen Belastungen der ehrenamtlich Tätigen können eine kontinuierliche Mitarbeit erschweren und manchmal fehlen tatsächlich Fachkenntnisse, um komplexe Studiendesigns zu verstehen [58]. Zudem besteht die Befürchtung, nicht ernst genommen oder für die Zwecke der Forschung instrumentalisiert zu werden, beispielsweise nur als Mittel zur Rekrutierung von Studienteilnehmenden [54]. Daneben ist vielen SHG- und SHO-Mitgliedern nicht bewusst, dass sie selbst über wertvolles kollektives Erfahrungswissen verfügen, welches sie sinnhaft und wirksam in Forschungsprojekte einbringen können. Vielfach fehlt es daher an Selbstverständnis und schlichtweg Mut, sich an Forschungsprojekten zu beteiligen. Auch sind die Wege in die Forschung für Interessierte oft unklar und Forschungsaufgaben erscheinen aus der Ferne zu komplex, um an ihnen mitwirken zu können. Diese und weitere Faktoren sind vermutlich die wesentlichen Gründe dafür, dass es schwer ist, Mitglieder von SHG und SHO für diese erweiterten Aufgaben zu aktivieren, wodurch viele Beteiligungsmöglichkeiten (z. B. in Gremien, Ausschüssen, Arbeitsgruppen) ungenutzt bleiben [9].

Ein Schlüssel zur Überwindung dieser Barrieren liegt in der gezielten Befähigung (Empowerment) und Ermutigung der SHG- und SHO-Mitglieder. Spezifische, bereits oben erwähnte Fortbildungsprogramme sind hilfreich für eine erfolgreiche Beteiligung. Aber auch den SHG und SHO selbst kommt eine Rolle in der Ermutigung ihrer Mitglieder zu. Wichtig ist, dass forschungserfahrene Mitglieder ihr Wissen über die Logiken und Praktiken der Wissenschaft weitergeben, Wege in die partizipative Forschung aufzeigen und diese ggf. in Form von Mentor:innenprogrammen oder Vertretungsregelungen gemeinsam gestalten.

Schließlich bleibt aber auch zu erwähnen, dass viele SHO in ihrer personellen Zusammensetzung durchaus mit hohen Fachkompetenzen aufwarten können. Eine Krebsdiagnose ereilt nun mal auch Ärzt:innen, Biolog:innen, Jurist:innen, Gesundheitswissenschaftler:innen, ITler:innen etc.

### Politische Rahmung.

Die Bewegung hin zu mehr Partizipation wird maßgeblich von politischen Initiativen und rechtlichen Rahmenbedingungen getragen. Die Nationale Dekade gegen Krebs (NDK) ist der zurzeit wohl wichtigste politische Treiber für die Patientenbeteiligung in der onkologischen Forschung in Deutschland. Durch die Gründung der „Allianz für Patientenbeteiligung“ [59] und die Veröffentlichung der „Prinzipien für eine erfolgreiche Patientenpartizipation in der Krebsforschung“ [57] wurde eine deutliche normative Setzung mit einem Handlungs- und Orientierungsrahmen geschaffen. Entscheidend ist, dass die NDK ihre Forderungen auch mit finanziellen Anreizen unterlegt. Die Förderrichtlinien des Bundesministeriums für Forschung, Technologie und Raumfahrt (BMFTR, früher BMBF) sehen in inzwischen verschärfter Form vor, dass nur noch Forschungsanträge berücksichtigt werden, die eine „weitreichende und ernstgemeinte Beteiligung von Patientinnen und Patienten vorsehen“ [29].

### Notwendigkeit finanzieller Regelungen.

Ein kritischer, aber oft vernachlässigter Aspekt für das Gelingen von Partizipation sind klare und unbürokratische finanzielle Regelungen (Aufwandsentschädigungen, Pauschalen, Stellenanteile), um sowohl das ehrenamtliche als auch das hauptamtliche Engagement zu würdigen und zu ermöglichen [3]. Die Implikation ist weitreichend: Ohne verbindliche, steuerrechtlich abgesicherte und einfach handhabbare Regelungen für die Erstattung von Kosten und die Zahlung von Pauschalen oder gegebenenfalls projektbezogenen Stellenanteilen bleibt das Engagement hauptsächlich von der individuellen finanziellen Leistungsfähigkeit der Ehrenamtlichen abhängig. Der zwar lakonische, aber deshalb nicht falsche Spruch: „Ein Ehrenamt muss man sich leisten können“, drückt aus, dass sich nur bestimmte Personen engagieren können, was dem Prinzip einer breiten und repräsentativen Beteiligung widerspricht. Die Etablierung von klaren finanziellen Regelungen ist notwendig, um die Nachhaltigkeit und Inklusivität einer selbsthilfeorientierten Forschung sicherzustellen. Zum anderen bliebe die hauptamtliche Beteiligung – sofern die SHG oder SHO denn überhaupt über hauptamtliche Mitarbeitende verfügt. Den meisten onkologischen SHG und SHO fehlt allerdings die finanzielle Basis, um eine mitunter zeitintensive Arbeit innerhalb von Forschungsbeteiligungen selbst zu finanzieren. Daher sollten Forschungsförderungsinstitutionen, wie z. B. das BMFTR, der Innovationsfonds des G‑BA, die DFG oder die DKH, in partizipativen Projekten und Forschungsgremien Stellenanteile oder Vergütungen für hauptamtlich Tätige insbesondere zur Koordination der Forschungsbeteiligung mit einkalkulieren.

## Diskussion und Ausblick

Die Analyse der Bedeutung und des Mehrwerts selbsthilfeorientierter Forschung in der Onkologie zeigt Potenziale, aber auch Herausforderungen. Zunächst ist festzuhalten, dass „selbsthilfeorientiert“ in diesem Zusammenhang kein geläufiger Begriff ist. Die spezifische Selbsthilfeorientierung versteckt sich oft im Konstrukt „Patientenbeteiligung und -vertretung“, welche allerdings überwiegend von SHO- und SHG-Mitgliedern und teilweise auch von Selbsthilfekontaktstellen umgesetzt werden. Selbsthilfeorientierte Forschung ist somit kein Nischenthema oder „nice to have“, sondern eine notwendige und logische Ergänzung zur hoch technologisierten biomedizinischen Forschung. Sie schließt Lücken zwischen der (Weiter‑)Entwicklung von Diagnostik und Therapie und einer patientenorientierten Krebsversorgung, indem sie patientenrelevante Behandlungs- und Versorgungsaspekte in den Mittelpunkt rückt.

Die Rolle der Selbsthilfe hat sich in Versorgung und Forschung zu einem aktiven und zunehmend professionalisierten Partner entwickelt. Dieser Prozess wird durch unterstützende politische Initiativen wie die Nationale Dekade gegen Krebs, Vorgaben in der Forschungsförderung, insbesondere durch das BMFTR, die DKH oder den Innovationsfonds des G‑BA, sowie auch institutionelle Strukturen wie Patientenbeiräte oder runde Tische der Selbsthilfe beschleunigt und verstetigt.

Ein besonders hervorzuhebender Mehrwert der selbsthilfeorientierten Forschung ist ihre systemische Wirkung. Sie manifestiert sich nicht in isolierten Vorteilen für einzelne Akteure, sondern in einer symbiotischen Wertschöpfungskette, die das gesamte Gesundheitssystem positiv beeinflusst. Sie reicht von für Versorgung und Patientenorientierung relevanteren Forschungsfragen über die Entwicklung praxistauglicherer Lösungen, die Stärkung der Gesundheitskompetenz von Krebs-Patient:innen bis hin zu (zumindest potenziellen) gesundheitsökonomischen Vorteilen durch eine bedarfsgerechtere und effizientere Versorgung.

Die Vision ist ein onkologisches Forschungs- und Versorgungssystem, in dem die „Patient:innenevidenz“ – das kollektive Erfahrungswissen der Betroffenen – als gleichberechtigte und unverzichtbare Säule neben der akademischen Evidenz anerkannt ist. In dieses integrative Konzept wären alle Versorgungsebenen und beteiligten Berufsgruppen einzubeziehen. Patient:innenseitig ist die organisierte Krebs-Selbsthilfe hierbei der wichtigste Akteur. Die meisten forschenden Krebszentren haben dies längst erkannt und sich den bestehenden Krebs-SHG und -SHO geöffnet und zugewandt. Die damit verbundenen Ansprüche umzusetzen und aufrechtzuerhalten ist allerdings mit Aufwendungen, Ressourcen und strukturellen wie kommunikativen Herausforderungen verbunden. Die Kooperation mit der Krebs-Selbsthilfe in der Forschung hat einen eigenen Stellenwert im Projektmanagement und bedarf somit eigener personeller und materieller Ressourcen. Die Einbindung von engagierten Patienten- und Selbsthilfevertretenden geht über das ehrenamtliche Engagement hinaus und sollte daher angemessen finanziell entschädigt werden.

## Footnotes

1 CAR-T-Zelltherapie: chimäre Antigen-Rezeptor-T-Zelltherapie – eine neue Form der Immuntherapie.

2 CALR-Mutationen: Mutationen im Gen für das Protein Calreticulin – fördern das Tumorwachstum.

3 Wird hier als Eigenname verwendet und deshalb nicht gegendert.

4 CK (Selbsthilfeforschung, Patientenvertretung), SH (Selbsthilfevertretung, Wissenschaft), OK (Patienten- und Selbsthilfevertretung), JW (Selbsthilfeforschung, onkologische Versorgung).

5 (cancer OR oncolog*) AND („patient cent*“ OR „patient orient*“ OR „patient involvement“) AND („self-help group“ OR „support group“ OR „self-help organi*“ OR „patient organi*“ OR „patient representat*“ OR „patient advoca*“) Germany.
